# No differences in the body fat after violating core bioelectrical impedance measurement assumptions

**DOI:** 10.1186/s12889-021-10552-y

**Published:** 2021-03-12

**Authors:** Arshdeep K. Randhawa, Veronica Jamnik, Michael D. T. Fung, Adam S. Fogel, Jennifer L. Kuk

**Affiliations:** Sherman Health Science Research Centre, School of Kinesiology and Health Science, York University, Rm 2002, 4700 Keele Street, Toronto, ON M3J 1P3 Canada

**Keywords:** Bioelectrical impedance, Obesity, BMI, Body fat, Impedance, BIA assumptions

## Abstract

**Objective:**

It is unclear to what degree acutely violating bioelectrical impedance analysis (BIA) measurement assumptions will alter the predicted percent fat mass (%FM) and whether this differs by sex or body mass index (BMI).

**Methods:**

%FM was assessed under control, dehydration, exercise, water, food intake and non-voided bladder conditions with three BIA devices (Tanita: BC-418, TBF-314, & Omron HBF-306CN) for men (*n* = 23, age: 24.0 ± 5.2 years old) and women (*n* = 17, age: 22.5 ± 3.4 years old) separately.

**Results:**

For all BIA devices, there were no differences in the %FM values between the control and the other conditions in men or women (− 1.9 to 0.4%, *p* > 0.05). Across the three devices and five conditions, 97% of %FM tests returned values within 5% of control (2 tests), and 86% of tests were within 2% of control despite violating an assumption. The errors were greatest with dehydration and women were more likely to have a %FM difference greater than 2% than men with dehydration using the hand-to-foot device (Tanita TBF-314: 59% versus 9%). There were no differences in %FM between control and the conditions when examined by BMI (overweight/obesity: − 2.8 to 0.1% and normal weight: − 1.7 to 0.5%; BMI*trial, *p* = 0.99).

**Conclusion:**

%FM estimates were similar despite acutely violating the preliminary measurement BIA assumptions across a range of different BMIs. The minor variations in %FM are smaller than what would be expected with day-to-day variability or weight loss intervention but may be larger in women than men.

## What is already known about this subject?

- Bioelectrical impedance analysis (BIA) devices use proprietary equations based on the relationship between total or segmental impedance and total body water to measure body composition.
- The BIA equations were developed using generally normal weight & healthy populations.
- The BIA equations follow certain assumptions related to proper hydration and fluid distribution prior measurements.

## What does this study add?

- Regardless of the BIA assumption violated, there were no differences in the impedance and percent fat mass values between the control and the five conditions. With all differences being less than what is expected with day-to-day variation.
- BMI categories were not associated with differences in the impedance and percent fat mass values between the control and the condition trials.
- Women tend to have larger variability in their percent fat mass measures with dehydration than men.

## Introduction

Bioelectrical impedance analysis (BIA) is a convenient, non-invasive and non-intrusive device for estimating body composition [1, 2]. The use of BIA devices to assess body composition is common in health and fitness facilities, occupational populations and research studies [1, 3–5]. BIA devices use proprietary or published equations based on the relationship between total or segmental impedance and total body water volume [6]. BIA equations for predicting body composition are based on the premise that when an alternating potential is applied to the body, the amount of current that passes through the conductive water-containing tissues is related with the amount of fat free mass (FFM).

BIA devices measure the impedance to the flow of electrical current to estimate body composition where higher or greater electrical impedance is correlated with higher fat mass [2, 6, 7]. These equations assume proper hydration and fluid distribution. Accordingly, the National Institute of Health (NIH) recommends avoiding BIA measurements when participants are dehydrated, within 4-h of food and beverage consumption, have a full bladder and within 12 h of moderate-to-strenuous exercise [8]. Although the preliminary measurement BIA assumptions are well known, they are often not optimally followed in practice, particularly in the general public and occupational populations including military, police and firefighters [3–5, 9–11]. To that end, whether violating these assumptions may alter how BIA predicts FFM and total body water has not been sufficiently investigated.

Different BIA devices may also be impacted differently by violating the preliminary BIA measurement assumptions. Impedance can be assessed using foot to foot, hand to foot and hand-held devices, and thus the tissues through which the main electrical current travels may differ between these devices. Most devices are single-frequency devices that use a frequency of 50 kHz passing between two different points via surface electrodes, but can vary by electrode characteristics (number, type and placement), electric current frequency (single or multiple frequencies) and body position at measurement [12]. Although BIA proposes certain assumptions, the impact of not adhering to those assumptions prior to the BIA assessment has not been sufficiently explored.

Finally, the impact of violating these preliminary measurement BIA assumptions may be influence by obesity status. The commonly used BIA published equations were developed using normal weight (18.5 to 24.9 kg/m^2^), and generally healthy populations [6, 7]. Some studies suggest that BIA analyses underestimate the percent fat mass (%FM) in individuals with overweight or obesity (≥25 kg/m^2^), and may be related to differences in fluid distribution, resistive and volume properties among various body tissues [13–15]. It is important to understand if violating these preliminary measurement BIA assumptions may result in greater discrepancies among those with greater obesity as this may have greater implications for assessing changes in body fat in this high-risk population.

Therefore, the primary aim of this study was to examine the effects of water intake, dehydration, food intake, exercise, and bladder voiding on acute BIA body composition and impedance measurements using three BIA devices. The second aim of the study is to see whether these effects differ by body mass index (BMI) categories (normal weight and overweight/obesity) We hypothesize that the effects of water and/or food intake, and not voiding the bladder would increase the total body water volume leading to decrease in impedance and underestimation of %FM. While the effects of dehydration and exercise would decrease the total body water volume leading to increase in impedance and overestimation of %FM. Additionally, the FM in individuals with obesity might be underestimated even further after violating any of the BIA guidelines.

## Methods

### Participants

Students and staff from York University were recruited via posters on campus and snowballing to participate in this study. Interested individuals mostly students were contacted through emails where the study objectives were further explained and questions about the visits answered. The inclusion criteria were: (a) age 18–70 years, (b) able to speak/read English, and (c) screened through Physical Activity and Readiness Questionnaire for Everyone (PAR-Q+) [16]. Of the 52 potential participants contacted, a total of 41 participants consented and completed the study.

Written informed consent was obtained by all participants and ethics approval was obtained from the Human Participation Review Sub- Committee, York University’s Ethic Review Board (certificate #: e2012–283).

### Measurement procedures

Anthropometric data was obtained on height, body mass, waist, hip, ankle, bicep, wrist and waist diameter. Height and body mass were measured using a wall mounted measuring tape and digital scale respectively. Waist circumference was obtained at the iliac crest as recommended by the NIH [17]. The BMI was determined using the following equation: body mass (kg)/height (meters)^2^ [17]. Participants completed a questionnaire on age, sex, education, ethnicity, fluid and food intake, and current medications. One woman was removed from all analyses as she had a large variability in body mass between visits.

### Protocol

All the participants were assessed by the following three validated single frequency BIA devices [18–21] in the same order: (1) Body Composition Analyzer, Model: BC-418 (hand-to-feet) (Tanita, Illinois, USA) (2) Digital Weight Scale, Model TBF-314 (foot-to-foot), (Tanita, Illinois, USA) and (3) Fat Loss Monitor, Model: HBF-306CN (hand-to-hand), (Omron, Kyoto, Japan). The two Tanita devices output total and regional body composition and impedance data while the Omron machine only outputs total percent fat mass.

#### Visit 1

At the first visit, participants were tested under three conditions *(water intake trial, non-voided bladder trial and exercise trial)* along with the control trial. Participants were instructed to drink 3 L of water the day prior to testing to ensure proper hydration [22]. In addition participants were instructed to (1) abstain from exercise on the day of the visit, (2) fast for 4–5 h prior to their visit and (3) not void their bladder for at least 2 h before the visit.

At the laboratory, participants were given 5 min to drink 1 L of water and then shortly after underwenta BIA measurement (*water intake trial*). After 30 min they had a BIA measurement with their bladder still unvoided (*non-voided bladder trial*). Within 30–40 min of ingesting water, the volume of stomach contents usually return to the original state before the water intake [23]. Participants then voided their bladder on a urine reagent test strip (10 LG Parameter Urine Reagent Strips, Craig Medical Distribution, CA, USA) to test urine specific gravity [24]. The following reference values were used to determine hydration status: 1–1.010 indicates relative hydration, and a value of 1.020 or greater indicates relative dehydration [25]. Once the hydration levels were reached (1–1.010 on the urine reagent test strip), the BIA assessment was repeated (*control trial*).

Participants were then asked to run/speed walk on a treadmill at a moderate intensity (50–70% of age predicted HR_max_ using 220-age) for 15 min and then underwent BIA measurements again (*exercise trial*). Following the exercise for 15 min, the BIA measurement was repeated.

All the participants followed the same order of BIA measurements started with water trial, non-voided bladder trial, control trial and then followed by exercise trial on Visit 1. The order was placed in order to keep the time between conditions consistent and limit any carry-over effect.

#### Visit 2

The second visit occurred 7 days after the first visit. Prior to coming to the laboratory for the second visit, particpants were asked to: (1) abstain from exercise on the day of the visit, (2) fast for 4–5 h prior to their visit and (3) not void their bladder for 2 h before the visit. In addition participants were instructed to not consume any fluid for 5–8 h prior to the assessment. Upon arrival, participants voided their bladder on a urine reagent test strip to ensure that they were dehydrated prior to BIA assessment. Once the dehydration level was ensured, the BIA measurement was taken (*dehydrated trial*).

Afterwards participants were given 30 min to consume a high fat meal ad libitum (325 g Dr. Oetker Ristorante Mozzarella Pizza (Kcal: 880, Fat: 44 g, CHO: 76 g, Protein: 36 g), Pringles Original (Per 16 chips, Kcal: 150, Fat: 9 g, CHO: 15 g, Protein: 1 g), and water. After confirming that participants had returned to adequate hydration status, we then measured BIA (*food intake trial*).

#### Visit 3

Participants underwent a Dual-energy X-ray Absorptiometry (DXA), total body composition assessment (bone mineral content, %FM, FFM) using a General Electric Lunar Prodigy (GE, USA). Although the validation of the BIA devices used have been demonstrated in other samples [18–21], we compared the estimated %FM from BIA with those obtained by DXA to confirm the validity of these devices in our study sample.

Skinfold measurements were measured three times using caliper (Harpenden Skinfold Caliper, Model: CE 0120) at the triceps, biceps, subscapular and suprailiac crest to estimate %FM. The %FM was calculated using Durnin JV and Womersley equation using the sum of skinfolds [26]**.**

### Statistical analysis

Statistical analysis was performed using SAS verion 9.4 (SAS Institute Inc., Cary, N.C., USA), with a level of statistical significance set at alpha < 0.05. Means and standard deviations (M ± SD) were used to describe sample characteristics. All analyses were stratified by sex. A repeated measures analysis of variance (ANOVA) was used to compare %FM and impedance between the BIA control trial and each of the conditions (water intake, dehydrated, food intake, exercise, and non-bladder voiding). The BIA %FM measures from each BIA machine were also compared to sum of skinfolds and DXA. Post hoc analysis using Tukey multiple comparison test was used to determine differences among BMI categories in their %FM and impedance variations among trials.

The proportion of individuals with absolute differences in %FM of greater than day to day variation (< 2%, 2–5% or > 5%) between trials was examined for sex and BMI categories differences by Chi-square tests with Bonferroni adjustment. Because of the low numbers of participants with errors over 2% for many of the conditions, only sex differences were reported.

Lastly, we conducted the multiple regression analyses to identify the relationship between changes in impedance and body mass with %FM. The standardized estimates (expressed per standard deviation) were used to facilitate comparisons between the impedance and body mass estimates.

## Results

The participant characteristics are shown in Table 1 for men and women separately. The BMI ranged from 20.2 to 37.8 kg/m^2^.

**Table 1:** Sample Characteristics by Sex

	Men	Women
Total Sample	*n* = 23	*n* = 17
Age (years)	24.0 ± 5.2	22.5 ± 3.4
BMI (kg/m^2^)	25.9 ± 3.5	22.8 ± 2.8*
BIA Body Fat (%)		
BC-418	19.7 ± 6.6	29.4 ± 6.9*
TBF-314	20.0 ± 6.8	27.1 ± 6.4*
Omron HBF	17.9 ± 6.7	24.4 ± 5.8*
DXA Body Fat (%)	20.7 ± 9.0	30.2 ± 8.5*
Skinfolds Body Fat (%)	19.1 ± 5.5	29.1 ± 5.4*
Waist Circumference (cm)	79.7 ± 15.5	76.2 ± 6.3
BIA Impedance (Ω)		
BC-418	560.2 ± 65.6	728.0 ± 88.8*
TBF-314	479.5 ± 52.7	581.3 ± 68.9*

All the continuous values are presented as means ± SD and categorical values as prevalence %

*BMI* Body mass index, * = significantly different from men (*p* < 0.05)

### Influence of various factors on BIA measures of percent fat mass

**Fig. 1:**
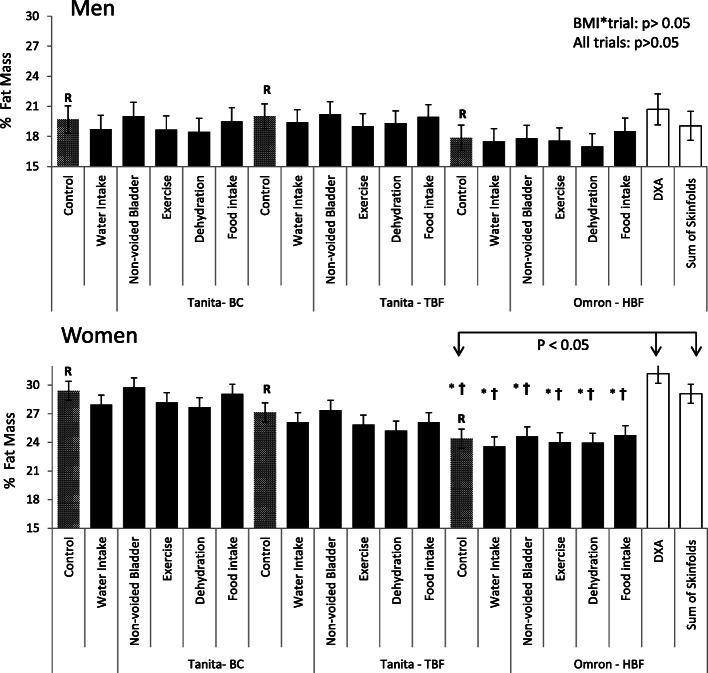
The average percent fat mass for each trial per BIA machine for men (*n* = 23) and women (*n* = 17). There were no differences between trials for each BIA machine for percent fat mass (*p* > 0.05). * = Significantly different from DXA and † = significantly different from sum of skinfolds

The %FM was assessed using three BIA devices (Tanita BC-418, Tanita TBF-314, and Omron HBF) under control, dehydrated, exercise, water and/or food intake, non-voided bladder conditions. These values are shown in Fig. 1 for men and women separately. For all BIA devices, there were no mean differences in the %FM values between the control and any of the condition trials in either men (range of means: − 1.2 to 0.3%) or women (range of means: − 1.9 to 0.4%, *p* > 0.05). Further, the differences in %FM between control and each condition trial was not significantly influenced by BMI categories (BMI*trial, *p* = 0.99).

### Influence of various factors on BIA measures of impedance

**Fig. 2:**
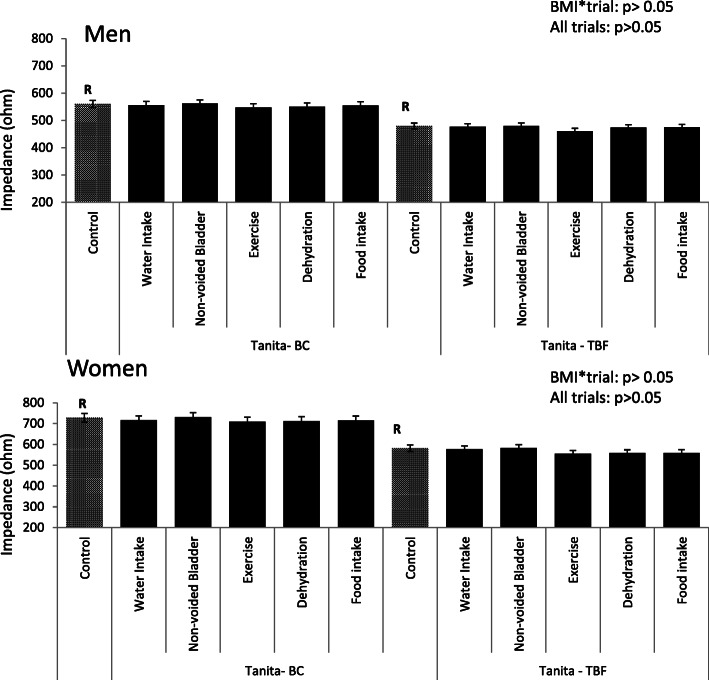
The average bioelectrical impedance for each condition per BIA Tanita machine for men (*n* = 23) and women (*n* = 17). There were no differences between conditions for each BIA machine for impedance from the reference group (*p* > 0.05)

Impedance tested using two BIA devices (Tanita BC-418, and Tanita TBF − 314) under various conditions (control, dehydrated, exercise, water and/or food intake, non-voided bladder) are shown in Fig. 2 for men and women separately. For both Tanita devices, there were no differences in the impedance values between the control and any of the condition trials (range: − 26.6 to 3.1 Ω, *p* > 0.05). Similar to %FM values, the differences in impedance between control and each condition trial was not significantly influenced by BMI (BMI*trial, *p* = 0.99).

In Table 2**,** the relationship between impedance and body mass with %FM in the control and condition trials are shown. The values of impedance and body mass for each condition are shown as the intra-individual difference between the control and the condition trial. During the control trial, total body impedance was more strongly related to %FM than body mass (standardized estimates; impedance, 5.13 to 8.48%, body mass, 4.89 to 5.59%). Similarly, we observed that the changes in total body impedance from the control trial were more strongly related with changes in %FM than changes in body mass for both Tanita BC-418 and TBF-314 **(**Table 2**)**. For example, one standard deviation change in impedance was associated with a 0.16 to 1.32% difference in FM while one standard deviation change in body mass was associated with a 0.22 to 0.79% difference in FM under various BIA conditions **(**Table 2**)**.

**Table 2:** Change in %FM with changes in impedance and body mass after violating the preliminary measurement BIA assumptions

Baseline	BIA	Total Body Impedance			Body Mass (BM)		
		Partial R	Parameter Estimate (%FM/Ω)	Standardized Estimate (%FM/SD)	Partial R	Parameter Estimate (%FM/kg)	Standardized Estimate (%FM/SD)
Control trial	BC-418	0.83	1.03	8.48%	0.70	0.68	5.59%
	TBF-314	0.59	0.69	5.13%	0.57	0.66	4.89%
Trial	**BIA**	**Change in %FM with Impedance**			**Change in %FM with BM**		
		Partial R	Parameter Estimate (%FM/Ω)	Standardized Estimate (%FM/SD)	Partial R	Parameter Estimate (%FM/kg)	Standardized Estimate (%FM/SD)
Water intake	BC-418	0.82	0.75	0.79	0.85	0.23	0.24
	TBF-314	0.81	0.53	0.34	0.85	0.62	0.40
Voided Bladder	BC-418	0.95	0.75	0.52	0.85	0.38	0.27
	TBF-314	0.75	0.54	0.16	0.83	0.72	0.22
Exercise	BC-418	0.85	0.80	0.55	0.55	0.32	0.22
	TBF-314	0.80	0.72	0.34	0.69	0.50	0.24
Dehydration	BC-418	0.92	0.90	1.31	0.81	0.54	0.79
	TBF-314	0.93	0.90	1.18	0.84	0.54	0.71
Food intake	BC-418	0.94	0.94	1.32	0.73	0.35	0.49
	TBF-314	0.92	0.94	1.13	0.66	0.35	0.42

Standardized estimates are expressed as %change in fat mass per one standard deviation change in impedance or body mass. The values of impedance and body weight for each condition were shown as the intra-individual difference between the control and the condition trial

When examining individual level data, across the three devices and five conditions, 97% of participants had differences in %FM that were within the expected day-to-day variation (< 5%) across all trials and BIA machines. Only two participants had variations in %FM that were greater than 5%, one normal weight woman participant when assessed using the Tanita BC-418 (5.8% lower %FM with dehydration) and one normal weight man participant when assessed using the Omron HBF (6.4% higher with water intake). Similarly, 86% of test conditions were within 2% variability of control condition.

**Fig. 3:**
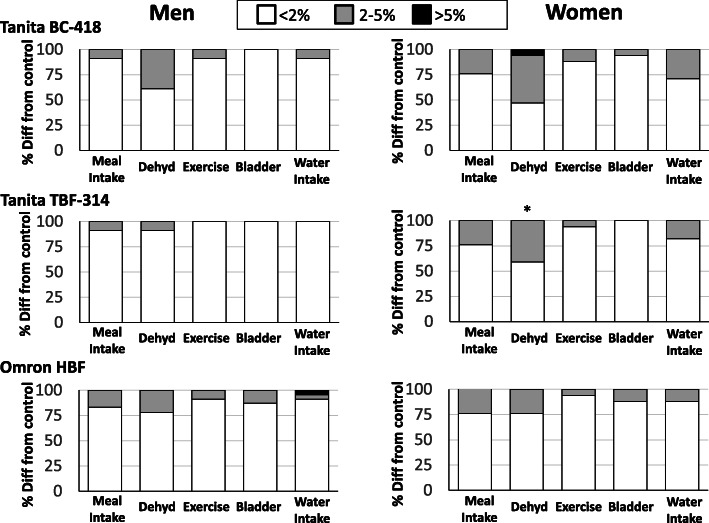
Prevalence of error rates by trial condition, sex and BIA device. *Sex difference in the prevalence of error < 2% within that device

Across the machines, bladder emptying and exercise having the least effect with over 95% of patients with differences of less than 2% while dehydration had the greatest variability with 68% of patients with less than 2% difference from control. Women were more likely to have an error of greater than 2% with dehydration than men when using the Tanita TBF-314 (Fig. 3: 59% versus 9%; *P* < 0.05). There were no differences by BMI (*P* > 0.05).

## Discussion

Our findings suggest that in our sample, acutely violating the preliminary measurement BIA assumptions does not significantly impact the derived %FM and impedance values. In general, women appear to have more variability in their BIA measures than men, particularly with dehydration using the hand-to-foot device. However, these minor differences in the measurements were similar among normal participants as compared to what was observed in those with overweight or mild obesity. The magnitude of the differences between trials are less than what is expected with day-to-day variation (< 5%) [21, 27].

In terms of water and food intake, the literature is inconsistent on the magnitude and even direction of change [28–32]. Similar to some studies [28, 29], we report non-statistically significant differences in %FM of ~ 1%. However, even in studies that report statistically significant differences with water and/or food intake, the magnitude of these differences are generally < 2% [21, 30, 31, 33, 34]. Similarly, a recent study by Ugras et al. [32] report that in dehydrated individuals, 500 ml of water intake is associated with a 2% increase in %FM when using a foot-to foot BIA device. Thus, water intake is likely associated with small differences in BIA measured %FM that is within ranges expected with day-to-day variation [21, 27].

Further, there were no consistent differences between studies that do or do not report significant differences in %FM in terms of diet composition, with high fat, high carbohydrate and ad libitum food intakes most commonly examined. The composition of the diet theoretically may influence body impedance and the rate of gastric emptying, however, one study reports that impedance values are similar immediately after consuming a meal and many hours later [29]. We extend previous observations by demonstrating that impedance measures after a large high fat meal of over 1000 kcal and water intake are not significantly different using either hand-to-hand, hand-to-foot or foot-to-foot BIA machines. Together, this suggests that food and/or water intake is unlikely to have a meaningful impact on impedance measured body fat assessment.

The non-voided bladder condition did not significantly change the impedance or %FM values when compared to the control trial. Although, the consumption of 1 L of water did increase body mass it was not enough to statistically increase %FM. In this study, 1 kg difference in body mass is theoretically associated with a 0.68% higher FM which is in line with a previous study that suggests a non-voided bladder could affect BIA measurements by up to 1% [35]. Thus, non-voided bladder is likely to have minimal effects on %FM estimates.

There are several changes that occur with exercise such as changes in skin blood flow, temperature, heat production and fluid loss [29], that may increase or decrease impedance. The literature on the effects of exercise on estimated %FM and impedance is mixed with studies showing decreased impedance by 28–40 Ω [36], or no change in impedance following moderate intensity aerobic exercise [36–38] as observed in this study. In the literature, the largest differences observed are less than 1% FM even with exercise intensity of 60 to 83% HR_max_ for as long as 45 min. These minimal differences suggest that moderate intensity exercise is unlikely be associated with large differences in predicted %FM.

For dehydration, theoretically one would expect low fluid status would result in an increase in impedance and thus increase in predicted %FM. In this study, impedance was not significantly increased in the dehydrated condition, and in fact trended in the opposite direction (Women: − 23.2%; Men: − 9.8%) and %FM (Women: − 1.9%; Men: 1.2%). The lower %FM is likely due to the reduction of average body mass of − 0.74 kg among participants. A study conducted by Thompson et al. [39] also report a significant decrease in %FM in the dehydrated state after exercising for 30 min and sitting in a steam room when compared with control, though the exact %FM difference was not reported. However, that study had a much larger decrease in body mass (average of 2.81%) than was observed in our study (< 1%). Further, we report that the differences in BIA measured %FM may be larger in women than men. Reasons for this are unclear but may be due to differences in %FM and fat distribution. That said, it is important to consider that we did not observe differences by BMI category. Despite our large range in BMI (20.2 to 37.8 kg/m^2^) the difference in %FM that resulted by violating the preliminary BIA assumptions are similar between BMI categories. Thus, future work may consider the potential sex differences in how these factors, and in particular dehydration may influence body composition assessment. Nevertheless, it is important to remember that the vast majority of values observed in this study were within 5% of control. Further, the < 2% differences we observe in this study are far lower than the 15 to 19.5% reduction in FM that are reported in exercise intervention even with minimal weight loss [40].

Further, these measures were generally comparable to DXA and sum of skinfolds assessments. The exception was the Omron HBF (hand-to-hand model) in women where the %FM values were significantly lower than DXA and sum of skinfolds. This reinforces the notion that %FM obtained cannot be directly compared between the various devices, but also suggests that the acute violation of the core BIA assumptions may not have a large influence on the %FM obtained regardless of the measurement site used. Further, these variations in %FM are far smaller than what one would expect with clinical weight loss interventions [41].

Some strengths and limitations of this study are worth mentioning. We are one of the few studies to examine the effect of violating the core BIA assumptions on the estimation of body composition among multiple BIA devices. In the current study, three BIA devices with different measurement sites were used. Although there are several different devices available on the market, they all use measures of impedance and body weight to assess body composition. That we also observe no differences in impedance suggest that these observations likely hold true for other BIA devices using different algorithms. However, we are unsure if the differences in body composition would be larger if more than one core BIA assumption was violated at the same time. We are also unsure if our results extend to older individuals or populations with chronic conditions. Finally, we have a relatively small sample of 40 adults, and retrospective power analyses suggest that 182 participants are needed for the largest difference (− 1.9%FM) to be significant. Nevertheless, the clinical relevance of these differences of this magnitude even if significant are questionable as they are comparable to be what would be expected with the 2 to 5% day-to-day variation [21, 27].

It can be concluded that preliminary measurement BIA assumptions have a very small effect (< 2%) on the derived %FM and impedance values. Women tend to have larger variability in %FM measures than men. Nevertheless, these differences associated with acutely violating the core BIA assumptions are far smaller than what would be expected with weight loss interventions and is within what is expected with day-to-day variation.

## Data Availability

The datasets during and/or analysed during the current study available from the corresponding author on reasonable request. Data will be safely stored in a locked facility and only research staff will have access to this information. Data will be kept for 10 years after the last publication, after which it will be securely destroyed or archived. Thus, we cannot ethically post the data publicly. However, with ethical approval, JLK would allow any requested data analyses.
